# Nitrate/ammonium-responsive microRNA-mRNA regulatory networks affect root system architecture in *Populus* × *canescens*

**DOI:** 10.1186/s12870-022-03482-3

**Published:** 2022-03-04

**Authors:** Jing Zhou, Jiang-Ting Wu

**Affiliations:** State Key Laboratory of Tree Genetics and Breeding, Key Laboratory of Silviculture of the National Forestry and Grassland Administration, Research Institute of Forestry, Chinese Academy of Forestry, Beijing, 100091 China

**Keywords:** miRNA, RNA-seq, Nitrogen, *P.* × *canescens*, Root system architecture

## Abstract

**Background:**

Nitrate (NO_3_^−^) and ammonium (NH_4_^+^) are the primary forms of inorganic nitrogen (N) taken up by plant roots, and a lack of these N sources commonly limits plant growth. To better understand how NO_3_^−^ and NH_4_^+^ differentially affect root system architecture, we analyzed the expression profiles of microRNAs and their targets in poplar roots treated with three forms of nitrogen S1 (NO_3_^−^), S2 (NH_4_NO_3_, normal), and S3 (NH_4_^+^) via RNA sequencing.

**Results:**

The results revealed a total of 709 miRNAs. Among them, 57 significantly differentially expressed miRNAs and 28 differentially expressed miRNA-target pairs showed correlated expression profiles in S1 vs. S2. Thirty-six significantly differentially expressed miRNAs and 12 differentially expressed miRNA-target pairs showed correlated expression profiles in S3 vs. S2. In particular, *NFYA3*, a target of upregulated ptc-miR169i and ptc-miR169b, was downregulated in S1 vs. S2, while *NFYA1*, a target of upregulated ptc-miR169b, was downregulated in S3 vs. S2 and probably played an important role in the changes in root morphology observed when the poplar plants were treated with different N forms. Furthermore, the miRNA-target pairs ptc-miR169i/b-*D6PKL2*, ptc-miR393a-5p-*AFB2*, ptc-miR6445a-*NAC14*, ptc-miR172d-*AP2*, csi-miR396a-5p_R + 1_1ss21GA-*EBP1*, ath-miR396b-5p_R + 1-*TPR4*, and ptc-miR166a/b/c-*ATHB-8* probably contributed to the changes in root morphology observed when poplar plants were treated with different N forms.

**Conclusions:**

These results demonstrate that differentially expressed miRNAs and their targets play an important role in the regulation of the poplar root system architecture by different N forms.

**Supplementary Information:**

The online version contains supplementary material available at 10.1186/s12870-022-03482-3.

## Background

Nitrogen (N) is one of the essential elements required by plants and plays key roles in their growth, development and morphological composition [1, 2]. In most higher plants, nitrate (NO_3_^−^) and ammonium (NH_4_^+^) are the main forms of inorganic N resources absorbed by plant roots from the soil [3, 4]. The mechanisms of NO_3_^−^ and NH_4_^+^ absorption and utilization differ among plants, and different N forms can have differential effects on plant root morphology [5–7]. In *Populus simonii* × *P. nigra* [5] and *Arabidopsis thaliana* [8], NH_4_^+^ can inhibit primary root growth compared to that observed in the presence of NO_3_^−^, whereas NH_4_^+^ has a greater promoting effect than NO_3_^−^ on lateral root development in *A. thaliana* [9]. Despite considerable research progress on the changes in root system architecture induced by different N forms [5, 8, 9], limited information is available regarding the underlying molecular mechanisms, especially the microRNA (miRNA) regulatory mechanisms underlying changes in root system architecture in the presence of different N forms.

miRNAs are highly conserved, endogenous, noncoding small RNAs with lengths of approximately 18–25 nucleotides. miRNAs inhibit posttranscriptional gene expression by inducing the cleavage of target genes or weakening translation, affecting organismal morphogenesis, development, and adaptability to environmental changes [10–14]. In recent years, increasing amounts of data have indicated that miRNAs are involved in herbaceous plant responses to various N stresses [6, 9, 15–17]. The first miRNA to be linked to the N response was miR167. In the pericycle cells of *Arabidopsis* roots, NO_3_^−^ (5 mM) inhibits miR167 expression while promoting the expression of its target *ARF8* (*AUXIN RESPONSE FACTOR 8*), which promotes lateral root initiation and emergence [9, 15]. In rice (*Oryza sativa*), miR166 targets *RDD1*(*rice Dof daily fluctuations 1*), which is related to NH_4_^+^ uptake and transport, leading to a change in root morphology [18]. These results, which were largely obtained in herbaceous plants, suggest that miRNAs are regulated by NO_3_^−^ and NH_4_^+^ and in turn regulate their target genes and affect the plant root system architecture [9, 15, 19]. Nevertheless, we have a poor understanding of the miRNA regulatory mechanisms affecting the root system architecture of woody plants under different N conditions.

Poplar, which presents a large demand for N fertilizer, has become a model system for research on the molecular mechanisms of woody plant root growth, development, and responses to the environment [2]. Some progress has been achieved in understanding the root morphological and physiological characteristics of several fast-growing poplar trees (such as *Populus* × *canescens* and *P. simonii* × *P. nigra*) as well as the regulation of distinct genes in the presence of different N forms [5]. Our previous studies have mainly focused on the role of miRNAs in association with different NO_3_^−^ absorption rates in different sections of poplar root tips [7]. However, few studies have focused on the links between morphological changes in roots and the expression levels of miRNAs and their targets when poplar plants are treated with different N forms (NO_3_^−^ and NH_4_^+^ or both).

In this study, using a hydroponic culture system, we monitored the root system architecture and molecular changes in *P.* × *canescens* when treated with 1 mM NO_3_^−^ (S1), 500 μM NH_4_NO_3_ (S2) or 1 mM NH_4_^+^ (S3) for 21 days. The primary goal of this work was to distinguish the morphological changes in poplar roots and analyze the potential miRNA-target pair regulation mechanisms of root morphological characteristics of poplar trees under different N forms. To achieve this goal, root length, lateral root initiation and lateral root density were measured. The results showed spatial variability in poplar root length and dry weight in the presence of different N forms. Through miRNA sequencing, degradome sequencing and transcriptomic sequencing, several candidate miRNA-target pairs associated with morphological changes in poplar roots were identified. The results of this work provide new ideas for the regulatory mechanisms whereby miRNA-mRNA regulatory networks change the root system architecture in response to different N forms, facilitating our understanding of the mechanism of root system architecture regulation in the presence of different N forms mediated by miRNA-target pairs.

## Results

### Morphological characteristics of P. × canescens roots in the presence of different N forms

**Fig. 1:**
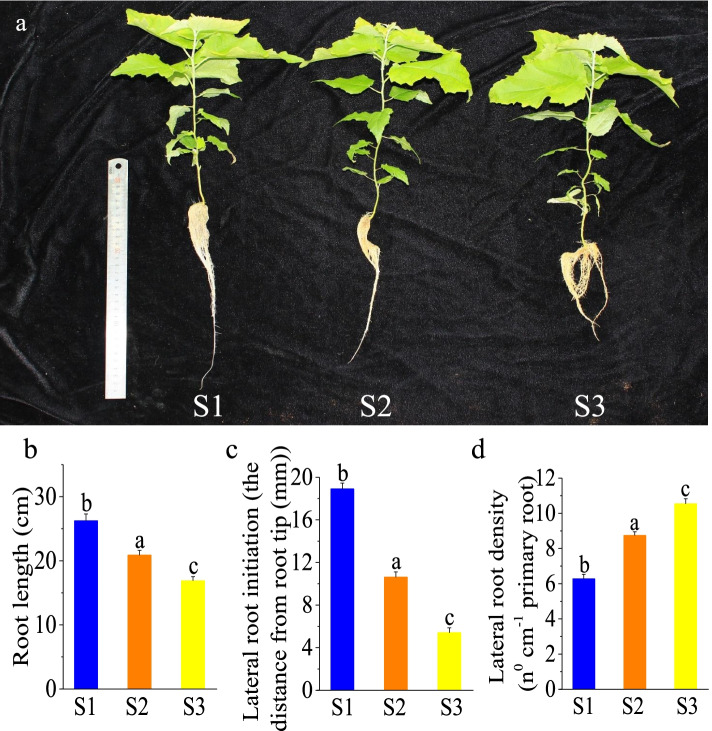
Morphological parameters of *P.* × *canescens* roots treated with different N forms for 21 days. Phenotypes of *P.* × *canescens* cultured with 1 mM NO_3_^−^, 500 μM NH_4_ NO_3_ or 1 mM NH_4_^+^ for 21 days (a). Root length (b), lateral root initiation (c) and lateral root density (d). Data are presented as the mean ± SEs (*n* = 18). a, b and c indicate significant differences based on one-way ANOVA and Duncan’s test (*P* < 0.05)

*P.* × *canescens* roots showed significantly different phenotypes after 21 days of long-term hydroponic cultivation with 1 mM NO_3_^−^ (S1), 500 μM NH_4_NO_3_ (S2, normal) or 1 mM NH_4_^+^ (S3). When the roots were grown in medium containing S1, root growth was significantly promoted compared to that in the presence of S2, whereas when the roots were grown in medium containing S3, root growth was significantly inhibited compared to that in the presence of S2. The length of the roots in S3 was 64% of that in S1 (Fig. 1a, b). The location of lateral root initiation in S1 was 19 mm from the root tip. The location of lateral root initiation in S2 was 10 mm from the root tip, and the location of lateral root initiation in S3 was 5 mm from the root tip (Fig. 1c). However, lateral root density was lower in S1 than in S2 and was lower in S2 than in S3 (Fig. 1d).

### Identification of novel and known miRNAs

Sequencing yielded approximately 11.96 million raw reads per library. Redundant reads were filtered to obtain clean reads. A total of 6 724 370, 5 799 345 and 5 653 606 valid reads corresponded to 2 066 888, 1 621 481 and 1 586 438 unique reads in the S1, S2 and S3 libraries, respectively (Supplementary Table 2). The sequences of low-quality and those ≤ 18 nt were removed, and the sequences of 18–25 nt were retained. The proportions of total and unique small RNAs (sRNAs) ranging from 18–25 nt are listed in Supplementary Fig. 1, among which sRNAs of 24 nts accounted for the highest proportion.

**Fig. 2:**
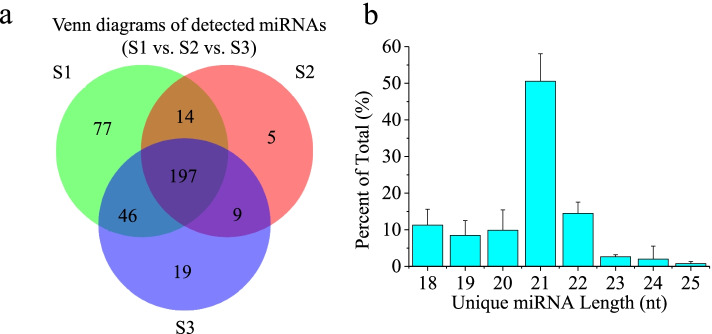
Venn diagrams of the detected miRNAs (**a**) and the lengths of the miRNAs (**b**) in three *P.* × *canescens* libraries

In total, 465 known miRNAs were identified in three libraries (Table 1, Supplementary Table 3), derived from 474 miRNA precursors belonging to 57 already documented miRNA families (Supplementary Table 4). Additionally, 29 novel miRNAs, corresponding to 26 miRNA precursors, were identified (Table 1, Supplementary Table 5). Among these miRNAs, 197 miRNAs were found in all three libraries, and 77, 5 and 19 miRNAs existed exclusively in S1, S2 and S3, respectively (Fig. 2a). The proportions of all identified miRNAs ranging from 18–25 nt are summarized in Fig. 2b, among which miRNAs of 21 nts accounted for the highest proportion.

**Table 1:** Number of identified known and novel miRNAs in *P.* × *canescens*

Samples	Known/novel miRNAs	Pre-miRNAs	Mature miRNAs
S1	known	394	373
	novel	16	17
S2	known	334	294
	novel	12	12
S3	known	346	319
	novel	17	19
Total	known	474	465
	novel	26	29

### Differentially expressed miRNAs in the presence of different N forms

**Fig. 3:**
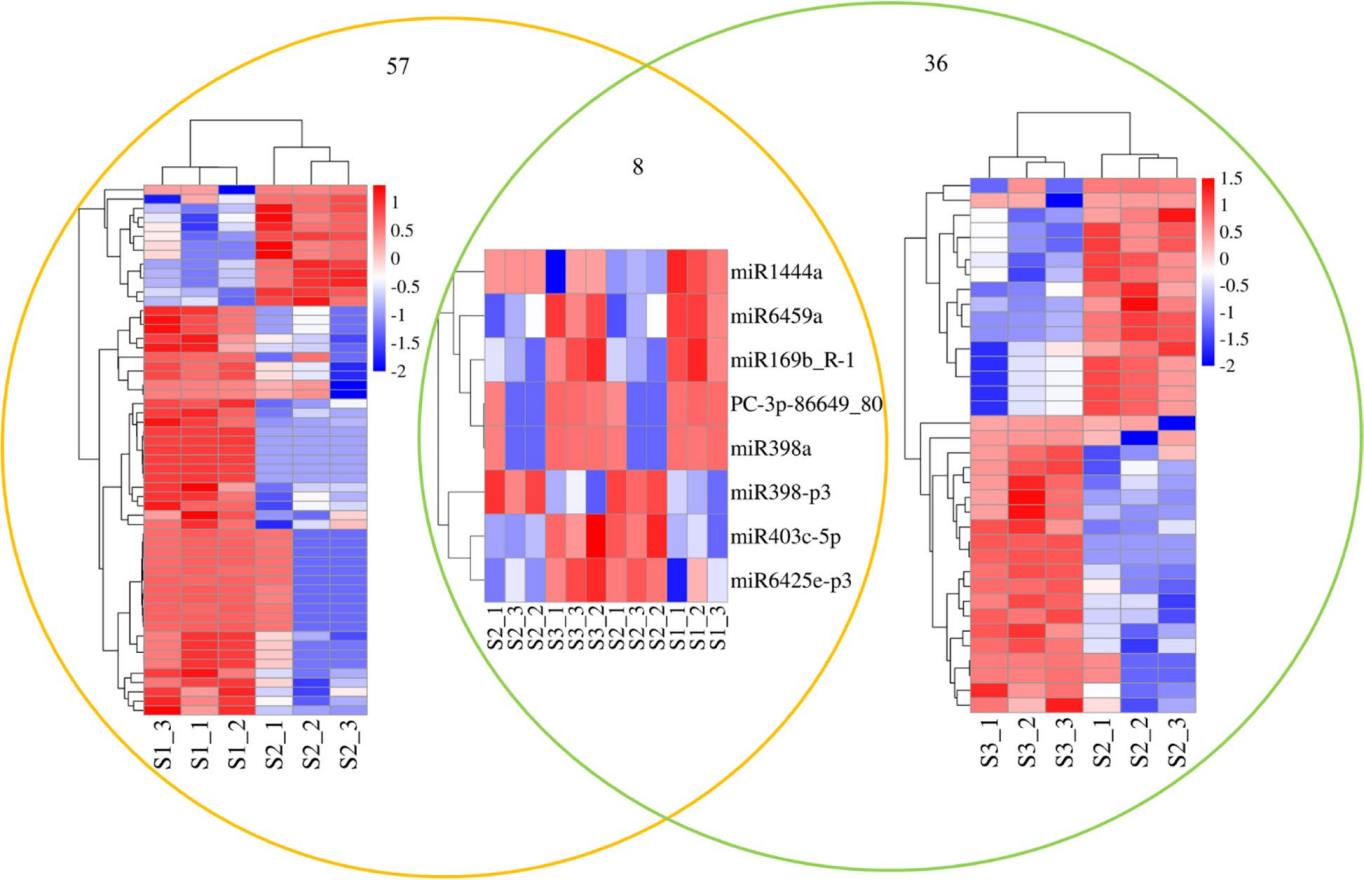
Significantly differentially expressed miRNAs in S1 vs. S2 and S3 vs. S2 of *P.* × *canescens*. Red and blue indicate up- and downregulated, respectively

In total, 54 known miRNAs belonging to 35 miRNA families and 3 novel miRNAs were significantly differentially expressed in S1 vs. S2 (Supplementary Table 6). Among these miRNAs, 44 upregulated and 13 downregulated miRNAs were identified in S1 vs. S2 (Fig. 3). The most upregulated miRNA identified in this comparison was PC-3p-86649_80 (15.13-fold), while the most downregulated was ptc-MIR169n-p5_2ss19TC21AC (5.79-fold). Additionally, 33 known miRNAs belonging to 19 miRNA families and 3 novel miRNAs were significantly differentially expressed in S3 vs. S2 (Supplementary Table 6). Twenty upregulated and sixteen downregulated miRNAs were identified in S3 vs. S2 (Fig. 3). Among them, the expression of PC-3p-86649_80 presented the greatest increase (9.05-fold), while the expression of ptc-miR6425a-5p showed the greatest decrease (10.66-fold).

We also compared the 57 significantly differentially expressed miRNAs in S1 vs. S2 with the 36 significantly differentially expressed miRNAs in S3 vs. S2 identified above, and the comparison is shown in Fig. 3. Eight significantly differentially expressed miRNAs were shared by both S1 vs. S2 and S3 vs. S2. Among the eight common significantly differentially expressed miRNAs, four (mdm-miR169b_R-1, ptc-miR398a, ptc-miR6459a-3p_R-1 and PC-3p-86649_80) were upregulated in both S1 vs. S2 and S3 vs. S2, one miRNA (sbi-MIR398-p3_2ss5GA18AG) was downregulated in both S1 vs. S2 and S3 vs. S2, one miRNA (ptc-miR1444a_R+1) was upregulated in S1 vs. S2 but had the opposite expression pattern (downregulated) in S3 vs. S2, and two miRNAs (ptc-miR403c-5p and ptc-MIR6425e-p3) were downregulated in S1 vs. S2 but had the opposite expression pattern (upregulated) in S3 vs. S2. These results suggest that the regulatory mechanisms of these five miRNAs could be conserved between S1 vs. S2 and S3 vs. S2, while sbi-MIR398-p3_2ss5GA18AG, ptc-miR403c-5p and ptc-MIR6425e-p3 may undergo a different evolution under treatment with different N forms.

### Validation of miRNAs sequence

To validate the miRNA sequence results, we selected twenty-two miRNAs in S1 vs. S2 and fifteen miRNAs in S3 vs. S2 for qRT–PCR (Supplementary Fig. 2). log_2_(FC) could be fitted by linear equations in both qRT–PCR and miRNA-seq. The slopes were 0.34 in S1 vs. S2 (Supplementary Fig. 2a) and -0.002 in S3 vs. S2 (Supplementary Fig. 2b). These values showed that log_2_(FC) in qRT–PCR was positively correlated with miRNA-seq in S1 vs. S2 and S3 vs. S2, indicating that miRNA-seq results were reliable.

### Prediction of miRNAs targets by degradome sequencing

To verify the targets of these miRNAs, three mixed degradome library sequencing analyses were performed to identify miRNA targets using the same total RNA samples. Through degradome sequencing, 78 470 784 raw reads representing 20 632 172 unique raw reads were generated from the three mixed degradome libraries. After the adapter-missing reads were removed, 78 109 423 (99.54% of all reads) sequences that were mapped to 6 812 869 unique transcript reads were successfully mapped against the *P.* × *canescens* transcriptomic data. According to the signature abundance of each occupied transcript site, all transcripts that could be cleaved were divided into five categories. A total of 1826 targets of 78 known miRNA families were identified from degradome sequencing in S1 vs. S2. We also identified 11 targets of novel miRNAs, which targeted 89 transcripts according to degradome sequencing in S1 vs. S2 (Supplementary Table 7). Additionally, a total of 1813 targets of 76 known miRNA families were identified from the degradome in S3 vs. S2. We also identified 8 targets of novel miRNAs, which included 79 transcripts according to degradome sequencing in S3 vs. S2 (Supplementary Table 7).

### Differentially expressed miRNAs and their targets involved in the regulation of the poplar root system architecture in the presence of different N forms

To better understand the functions of significantly differentially expressed miRNAs, we predicted 227 and 126 target genes of these miRNAs in S1 vs. S2 and S3 vs. S2, respectively (Supplementary Table 8). MapMan was used to assign these targets to functional categories (Supplementary Table 8). Several functional categories, including RNA regulation of transport, transcription and development, were associated with the regulation of the poplar root system architecture in the presence of different N forms (Supplementary Table 8). Among the identified target genes, *AAP7* (an *amino acid transmembrane transporter*), which is targeted by gra-MIR8654c-p3_2ss12TG18AG family members, is responsible for the transport of amino acids. Several targets, including *NFYA1/3* (*nuclear transcription factor Y subunit A*), *ARFs* (*auxin response factors*), *AP2/EREBP* (*APETALA2/ethylene-responsive element binding protein*) family members and *NAC* (*NAC transcription factor*) transcription factors, which belong to the miR169, miR160, miR172 and miR6445 families, accounted for the largest proportion of targets involved in the RNA-mediated regulation of transcription (Supplementary Table 8). These transcription factors are closely related to plant development and the response to nitrogen treatments [7, 9, 15, 20–23].

The functions of these 227 and 126 targets were further revealed by GO functional classification analysis. These identified target genes are predominantly involved in biological processes, cellular components and molecular functions. Twenty-five biological processes were identified, with the most frequent category being ‘transcription, DNA-templated’. Among the 15 cellular component categories, the most representative was ‘nucleus’. Finally, there were 10 molecular function categories, with the most abundant being ‘sequence-specific DNA binding transcription factor activity’ in S1 vs. S2 and S3 vs. S2 (Fig. S3a, b). This finding suggested that *P.* × *canescens* adopted complex and broad responsive approaches to accommodate the challenges of different N form conditions. KEGG analysis showed that the maximum target gene categories were differentially expressed for plant signal transduction pathways in both S1 vs. S2 and S3 vs. S2 (Fig. S4a, b). These pathways could be related to plant N physiological processes and root growth or development.

### Correlations between miRNAs and their targets

**Fig. 4:**
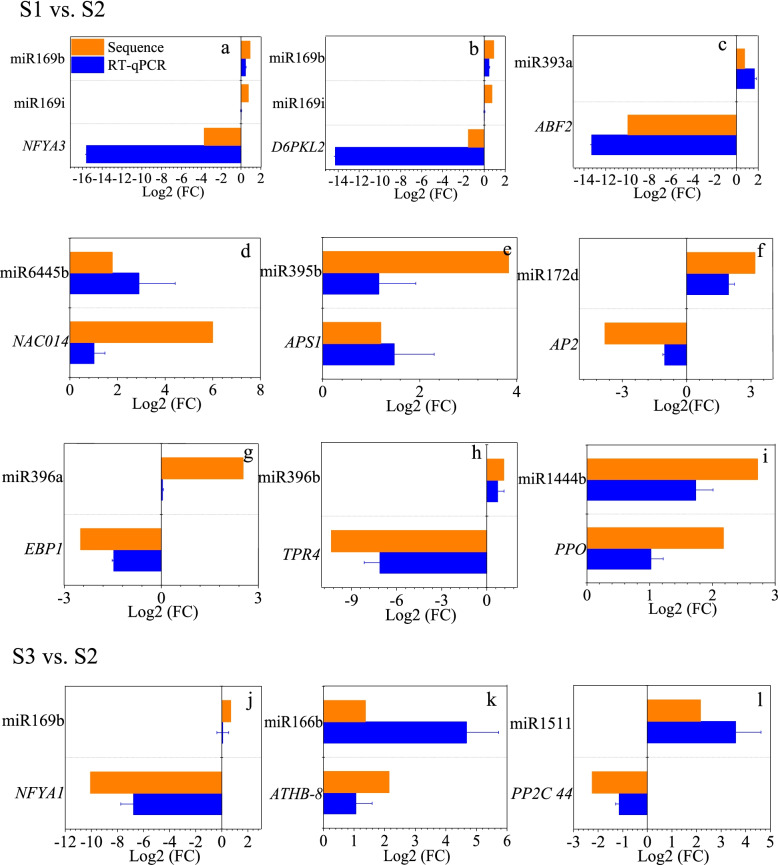
Validation of significantly differentially expressed miRNAs and their targets in *P.* × *canescens* in the presence of different N forms by sRNA-seq and qRT–PCR

Twenty-eight significantly differentially expressed miRNA-target pairs were identified in S1 vs. S2 (Table 2). Among them, seventeen miRNA-target pairs were negatively correlated. In contrast, the expression levels of the other 11 miRNA-target pairs were positively correlated. Meanwhile, twelve significantly differentially expressed miRNA-target pairs were identified in S3 vs. S2 (Table 2). The expression levels of 4 miRNA-target pairs displayed negative correlations. In contrast, the expression levels of the other 8 miRNA-target pairs showed positive correlations. The correlations between these miRNAs and their targets were verified by qRT–PCR (Fig. 4).

**Table 2:** The differentially expressed targets of N-responsive differentially expressed miRNAs

	miR_name	Up/down	Targets	log_2_(FC)	annotation
S1 vs. S2	ptc-miR169i_1ss15TA	up	Potri.018G064700.2	-3.71	*nuclear transcription factor Y subunit A-3 (NFYA3)*
	mdm-miR169b_R-1	up	Potri.018G064700.2	-3.71	*nuclear transcription factor Y subunit A-3 (NFYA3)*
	mdm-miR169b_R-1	up	Potri.017G075400.1	-1.52	*serine/threonine-protein kinase AtPK2/AtPK19-like (D6PKL2)*
	ptc-miR169i_1ss15TA	up	Potri.017G075400.1	-1.52	*serine/threonine-protein kinase AtPK2/AtPK19-like (D6PKL2)*
	ptc-miR393a-5p	up	Potri.001G323100.1	-9.97	*Protein AUXIN SIGNALING F-BOX 2 (AFB2)*
	mes-MIR393b-p3_1ss21AG	up	Potri.011G060800.1	1.66	*28 kDa heat- and acid-stable phosphoprotein (PDAP1)*
	ptc-miR6445a	up	Potri.013G079700.3	5.99	*NAC domain-containing protein 14 (NAC014)*
	ptc-miR6445a	up	Potri.013G079700.4	12.05	*NAC domain-containing protein 14 (NAC014)*
	ptc-miR6445a	up	Potri.013G079700.8	3.27	*NAC domain-containing protein 14 (NAC014)*
	ptc-miR395b	up	Potri.008G159000.3	1.20	*ATP sulfurylase family protein (APS1)*
	ptc-miR395b	up	Potri.008G159000.1	2.65	*ATP sulfurylase family protein (APS1)*
	ptc-miR172d	up	Potri.007G046200.2	-3.20	*Floral homeotic protein APETALA 2 (AP2)*
	ptc-miR172d	up	Potri.007G046200.1	-3.81	*Floral homeotic protein APETALA 2 (AP2)*
	ptc-miR172d	up	Potri.005G140700.2	-1.96	*Floral homeotic protein APETALA 2 (AP2)*
	csi-miR396a-5p_R + 1_1ss21GA	up	Potri.006G102200.4	-12.2	*ERBB-3 BINDING PROTEIN 1 (EBP1)*
	csi-miR396a-5p_R + 1_1ss21GA	up	Potri.006G102200.3	-2.50	*ERBB-3 BINDING PROTEIN 1 (EBP1)*
	ama-miR396-5p_R + 1_1ss19CT	up	Potri.002G082400.1	1.73	*IAA-amino acid hydrolase ILR1-like 6 (ILL6)*
	ath-miR396b-5p_R + 1	up	Potri.006G066800.1	-10.34	*topless-related protein 4-like (TPR4)*
	ptc-MIR1444b-p3	up	Potri.T062200.1	2.03	*polyphenol oxidase, chloroplastic-like (PPO)*
	ptc-MIR1444b-p3	up	Potri.T061900.1	2.32	*polyphenol oxidase, chloroplastic (PPO)*
	ptc-MIR1444b-p3	up	Potri.001G388800.1	1.77	*polyphenol oxidase, chloroplastic-like (PPO)*
	ptc-MIR1444b-p3	up	Potri.001G387900.1	2.14	*Polyphenol oxidase, chloroplastic (PE)*
	ptc-MIR475c-p5	up	Potri.006G259400.5	-13.3	*Homeobox protein knotted-1-like 3 (KNAT3)*
	aly-miR159c-3p_R + 1_1ss20CT	up	Potri.002G001900.2	-6.80	*protein ternary complex factor MIP1*
	ptc-MIR6462f-p3_1ss16TC	up	Potri.001G422300.1	-1.22	*-*
	ptc-MIR6462d-p3_1ss7AG	up	Potri.001G422300.1	-1.22	*-*
	ptc-MIR6462a-p3_1ss7AG	up	Potri.001G422300.1	-1.22	*-*
	mtr-MIR2592bj-p3_1ss12TC	down	Potri.002G003600.4	1.00	*PREDICTED: DEAD-box ATP-dependent RNA helicase 42-like*
S3 vs. S2	mdm-miR169b_R-1	up	Potri.009G060600.1	-10.1	*Nuclear transcription factor Y subunit A-1 (NFYA1)*
	gma-miR6300	down	Potri.007G138600.1	-1.59	*Cell cycle regulated microtubule associated protein (TPX2)*
	gma-miR6300_R + 1_2	down	Potri.007G138600.1	-1.59	*Cell cycle regulated microtubule associated protein (TPX2)*
	gma-miR1511_R-2	up	Potri.004G127400.3	-1.20	*-*
	gma-miR1511_R-2	up	Potri.008G168300.5	-2.27	*protein phosphatase 2C-like protein 44*
	stu-miR166b_R-1_1ss19CT	up	Potri.007G119200.2	-1.72	*eukaryotic translation initiation factor (eIF)*
	stu-miR166b_R-1_1ss19CT	up	Potri.018G045100.3	2.18	*Homeobox-leucine zipper protein ATHB-8 (ATHB-8)*
	mtr-miR166c_2ss20TC21CT	up	Potri.018G045100.3	2.18	*Homeobox-leucine zipper protein ATHB-8 (ATHB-8)*
	ptc-miR166a	up	Potri.018G045100.3	2.18	*Homeobox-leucine zipper protein ATHB-8 (ATHB-8)*
	mtr-miR166c_2ss20TC21CT	up	Potri.018G045100.1	1.09	*Homeobox-leucine zipper protein ATHB-8 (ATHB-8)*
	stu-miR166b_R-1_1ss19CT	up	Potri.018G045100.1	1.09	*Homeobox-leucine zipper protein ATHB-8 (ATHB-8)*
	ptc-miR166a	up	Potri.018G045100.1	1.09	*Homeobox-leucine zipper protein ATHB-8 (ATHB-8)*

### Validation of the miRNA-target pairs

**Fig. 5:**
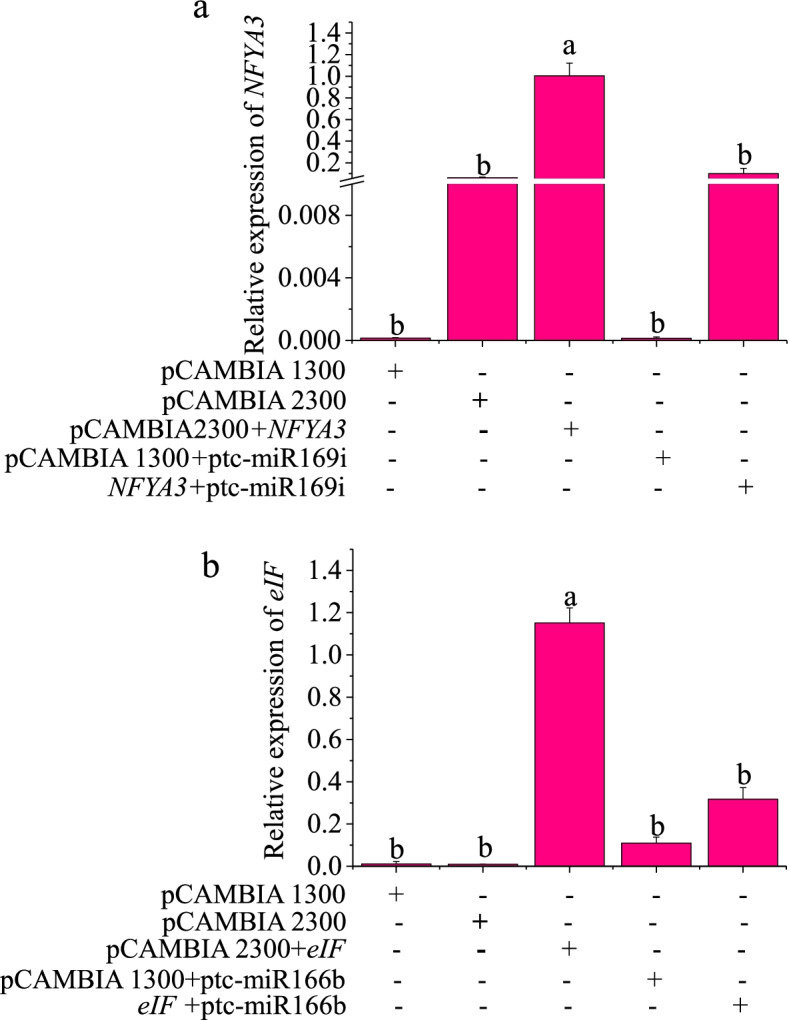
Degradation effect of miRNAs on target genes. In the graph, validation of ptc-miR169i-*NFYA3* (**a**) and ptc-miR166b-*eIF* (**b**) using transient coexpression assays in *N. benthamiana* leaves. The expression levels were quantified using qRT–PCR. Bars indicate means ± SE (*n* = 4). Different letters on the error bars indicate significant differences

To validate the miRNA-target pairs predicted via degradome sequencing, transient coexpression experiments were performed in *Nicotiana benthamiana* leaves. Two randomly selected miRNA-mRNA pairs ptc-miR169i_1ss15TA and its target *NFYA3* (*Potri.018G064700.2*) and stu-miR166b_R-1_1ss19CT and its target *eIF* (*eukaryotic translation initiation factor*) (*Potri.007G119200.2*) were selected for transient coexpression experiments. The *NFYA3* transcript level was significantly decreased after *NFYA3* and ptc-miR169i_1ss15TA coexpression in *N. benthamiana* leaves for 2 days, and the expression of the target gene *eIF* was significantly decreased under *eIF* and stu-miR166b_R-1_1ss19CT coexpression; thus, the expression levels of the target genes *NFYA3* and *eIF* were decreased considerably by ptc-miR169i_1ss15TA and stu-miR166b_R-1_1ss19CT, respectively, in comparison with the expression levels observed under transient expression of the *NFYA3* and *eIF* target genes alone (Fig. 5). These results suggest that *NFYA3* and *eIF* are the targets of ptc-miR169i_1ss15TA and miR166b_R-1_1ss19CT, respectively.

### miRNA-target pairs involved in the regulation of the poplar root system architecture in the presence of different N forms

**Fig. 6:**
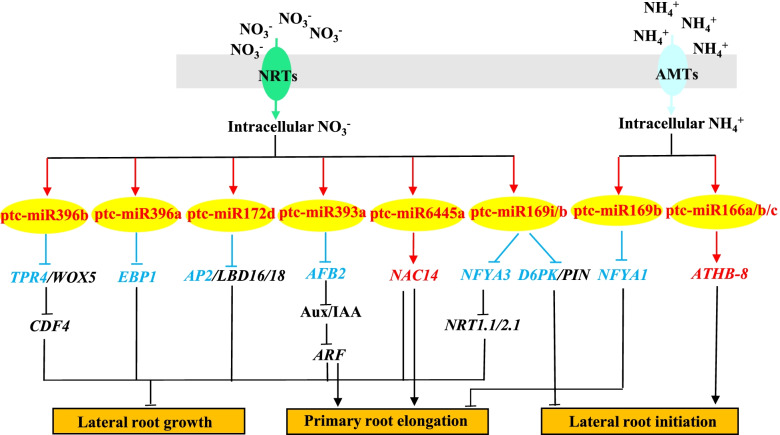
A simple model illustrating the effect of miRNA-target pairs on the morphological changes of poplar roots under treatment with different N forms

A BLASTN search of *Arabidopsis* and *P.* × *canescens* genomes was performed to annotate these significantly differentially expressed target genes of significantly differentially expressed miRNAs in S1 vs. S2 and S3 vs. S2 (Supplementary Table 9). We examined their annotation and found that nine miRNA-target pairs in S1 vs. S2 were strongly associated with the regulation of the poplar root system architecture (Fig. 6). These miRNA target pairs included ptc-miR169i/b and their target *NFYA3*, ptc-miR169i/b and their target *D6PKL2* (*serine/threonine-protein kinase D6PKL2*), ptc-miR393a-5p and its target *AFB2* (*AUXIN SIGNALING F-BOX2*), ptc-miR6445a and its target *NAC14*, ptc-miR172d and its target *AP2*, ath-miR396b-5p_R + 1 and its target *TPR4* (*topless-related protein 4-like*), and csi-miR396a-5p_R + 1_1ss21GA and its target *EBP1* (*ERBB-3 BINDING PROTEIN 1*). Four miRNA-target pairs in S3 vs. S2 had close relationships with the regulation of the poplar root system architecture (Fig. 6). These miRNA target pairs included ptc-miR169b and its target *NFYA1* and ptc-miR166a/b/c and their target *ATHB-8* (*homeobox-leucine zipper protein ATHB-8*). In particular, *NFYA3*, a target of upregulated ptc-miR169i and ptc-miR169b, was downregulated in S1 vs. S2, while *NFYA1*, a target of upregulated ptc-miR169b, was downregulated in S3 vs. S2, indicating that miR169-*NFYA* pairs probably had an important and complex regulatory effect on poplar root system architecture under different N forms.

## Discussion

### NO_3_^−^ treatment causes lateral root elongation, while NH_4_^+^ treatment tends to increase lateral root initiation and differentiation

Plant roots display high plasticity in response to different N forms, and the morphological characteristics of plant roots are distinctly different when NO_3_^−^ or NH_4_^+^ is used as the sole N source [5, 6, 24]. Previous studies have shown that the supply of NO_3_^−^ to plants mainly promotes the elongation of lateral roots, whereas the supply of NH_4_^+^ increases the initiation and branching of lateral roots [5, 25, 26]. For example, the root length of NO_3_^−^- or NH_4_NO_3_-treated *P. simonii* × *P. nigra* seedlings was greater than that of NH_4_^+^-treated seedlings after 21 days of treatment [5]. In *Arabidopsis*, the local supply of NH_4_^+^ increased lateral root initiation and lateral root branching [27, 28], in accord with our results. In this study, the root lengths of NO_3_^−^ - or NH_4_NO_3_-treated roots were greater than those of NH_4_^+^-treated plants, and NH_4_^+^ treatment resulted in earlier lateral root initiation than that observed under NO_3_^−^ treatment. These results suggest that poplar roots display high plasticity in response to different N forms. We propose that the greater root length observed under the influence of NO_3_^−^ could be a result of the increased supply of NO_3_^−^ due to the upregulation of nitrate transporters (NRTs) in NO_3_^−^-treated roots (S3), since NRTs are NO_3_^−^ transporters that enhance nitrate-dependent root elongation [14]. On the other hand, single or excessive NH_4_^+^ treatment can have adverse effects on plants, including altered root metabolism, plant ion imbalances, and foliar oxidative stress [29], leading to the growth and development of shorter roots. Liu et al. also speculated that high concentrations of NH_4_^+^ supplied as the sole nitrogen source would cause toxic symptoms and inhibit root growth in many plant species [8]. This may be the reason for the growth of shorter roots after NH_4_^+^ treatment.

### miRNAs can regulate targets participating in the uptake and assimilation of different N forms to induce changes in root development, including that of lateral roots

The morphological changes caused by different N forms are closely related to changes at the molecular level. To better understand the effects of different N forms on root morphology and growth, we detected the differentially expressed miRNAs and their targets in poplar roots treated with different N forms. In this study, 57 and 36 miRNAs showed differential expression patterns in the NO_3_^−^ and NH_4_^+^ libraries, respectively. These results suggested that NO_3_^−^ treatment elicits stronger miRNA responses than NH_4_^+^ treatment. The difference may reflect the fact that NO_3_^−^ supplied during long-term cultivation (21 days) can trigger a markedly different miRNA response in *P.* × *canescens*, which is consistent with previous research in rice [6]. In rice, the number of differentially expressed miRNAs identified in long-term NO_3_^−^-treated plants (16 miRNAs) was greater than that in NH_4_^+^-treated plants (11 miRNAs) [6]. As expected, the number of differentially expressed target genes observed under NO_3_^−^ treatment was greater than that under NH_4_^+^ treatment (data not shown), which was consistent with previous findings in *P. simonii* × *P. nigra* [5]. The 227 differentially expressed genes identified by RNA-seq under NO_3_^−^ treatment were greater than the 126 differentially expressed genes identified under NH_4_^+^ treatment in *P. simonii* × *P. nigra* [5]. Considering that NO_3_^−^ is an important signaling molecule [30–32], it is reasonable to speculate that the effect of NO_3_^−^ treatment on miRNA expression may be more pronounced than that of NH_4_^+^ treatment.

Some miRNA-target pairs were also involved in the changes in plant root morphology observed in the presence of different N forms [6, 7]. Several studies have shown that miR169 family members and their target *NFYA* transcripts play a role in the control of root growth in *A. thaliana* [22, 33], wheat [34], maize [35] and poplar [36]. In wheat, the overexpression of *TaNFYA*-*B1* promoted the expression of *TANRT1.1* and *TANRT2.1*, increased root NO_3_^−^ influx and promoted lateral root growth [34]. *NFYA3*, which is homologous to *TaNFYA*-*B1*, showed a significantly downregulated expression profile in S1 vs. S2 and inhibited lateral root growth. In rice, miR169o-overexpressing plants are taller than WT plants under either normal or limiting NO_3_^−^ conditions, and the predicted *OsNFYA1/4* target genes display an exactly opposite expression pattern to that of osa-miR169o in response to NO_3_^−^ deficiency [14]. *NFYA1* was homologous to *OsNFYA4*, and it showed a significantly downregulated expression profile in S3 vs. S2 and inhibited primary root elongation. These results indicate that ptc-miR169 family members and their target *NFYA* genes might participate in the alteration of *poplar* root morphology in response to different N forms. Interestingly, the expression of ptc-miR169i_1ss15TA and ptc-miR169b_R-1 exhibited another target, *D6PKL2*, in S1 vs. S2. D6PKL2 kinases may directly phosphorylate PIN-FORMED (PIN) proteins [37], and *d6pk mutants* show defects in lateral root initiation, and this phenotype is correlated with a reduction in auxin transport in *Arabidopsis* [38]. These results suggest that members of the same miRNA family may have different targets that perform different functions affecting root morphology in response to different N forms in *P.* × *canescens*. Thus, a complex mechanism may modulate the expression profiles of miRNA family members and their targets.

miR393 targets transcripts that encode the auxin receptors *TIR1* (*transport inhibitor response protein 1*), *AFB1*, *AFB2*, and *AFB3*. *AtAFB3* is induced by NO_3_^−^ in *Arabidopsis* root organs, which inhibits primary root elongation and promotes lateral root initiation [19]. In our research, *AFB2*, which is homologous to an *AtAFB3* sequence, was reduced in root organs after 21 days of exposure to NO_3_^−^ treatment, promoting primary root elongation and inhibiting lateral root initiation. This result differed from previous findings in *Arabidopsis* [19], possibly for the following reasons. First, compared with the *Arabidopsis* experiment, the treatment environment and conditions were different in this experiment. Second, the *AFB* gene family has different functions in annual herbs and perennial woody plants. Third, in *Arabidopsis*, *AFB3* was induced after 1 h of NO_3_^−^ treatment, but miR393 was also induced after NO_3_^−^ treatment, which led to the degradation of its target gene *AFB3* and reset *AFB3* expression to basal levels. Therefore, *AFB3* was induced by NO_3_^−^ treatment and degraded by miR393 to achieve negative feedback regulation [19]. However, in poplar trees, whether the miR393-*AFB2* pair has a negative feedback regulation mechanism under NO_3_^−^ treatment needs to be further studied.

The target gene of ptc-miR6445a is the *NAC14* transcription factor. A poplar homologous gene of *AtNAC4* acts downstream of *AFB3* to control the root response to NO_3_^−^, while *nac4* mutants exhibit changes in lateral root growth in *A. thaliana* [39, 40]. In wheat, *TaNAC2-5A* can be directly bound to the promoter regions encoding nitrate transporters and glutamine synthetase genes, which affects root growth and the nitrate influx rate [41]. ptc-miR172d was markedly upregulated in S1 vs. S2. The target gene of ptc-miR172d was the *AP2/ERF* transcription factor, a poplar homologous gene of *AtAP2/EREBP* involved in lateral root formation in *Arabidopsis* [42]. The target of csi-miR396a-5p_R + 1_1ss21GA was *EBP1*. A poplar homologous gene of *AtEBP1* is involved in dividing tissues during root development in *Arabidopsis* [43]. Another member of the miR396 family, ath-miR396b-5p_R + 1, shows the target gene *TPR4.* A poplar homologous gene of *AtTPR4* was recruited by *WUSCHEL RELATED HOMEOBOX 5* (*WOX5*) to inhibit target genes that promote differentiation to ensure the maintenance of stem cells [44, 45]. These results suggest that the csi-miR396a-5p_R + 1_1ss21GA-*EBP1* and ath-miR396b-5p_R + 1-*TPR4* miRNA-target pairs might participate in changes in *P.* × *canescens* root morphology by regulating the cell differentiation involved in NO_3_^−^ treatment. The target of ptc-miR166a/b/c was *ATHB8*, and both miR166 and the target gene *ATHB8* were upregulated in response to NH_4_^+^ treatment. A poplar homologous gene of *AtATHB8* affects vascular development [46] and may be related to lateral root initiation and differentiation [46, 47].

## Conclusions

Above all, these results identified several miRNA-target pairs that are involved in the assimilation of different N forms in *P.* × *canescens* and probably play important roles in poplar root morphogenesis. According to the present results, we propose a simple model of the response to different N forms in *P.* × *canescens* roots (Fig. 6). There were significant differences in the morphological characteristics of poplar roots among the three N form treatments after 21 days. Our global sRNA and transcriptome analysis showed that different N forms induce distinct miRNA-target pairs to regulate poplar root morphology, and regulation by these miRNA-target pairs forms the basis for the adaptation of poplar root morphology to different N forms. Notably, the ptc-miR169i/b-*NFYA1/3*, ptc-miR169i/b-*D6PKL2*, ptc-miR393a-5p-*AFB2*, ptc-miR6445a-*NAC14*, ptc-miR172d-*AP2*, csi-miR396a-5p_R + 1_1ss21GA-*EBP1*, ath-miR396b-5p_R + 1-*TPR4*, and ptc-miR166a/b/c-*ATHB-8* pairs probably play key roles in the changes in poplar root morphology under different N forms. In future works, we suggest the selection of functional target genes associated with root morphology and functional assignment of the transcription factors identified under treatment with different N forms. These results indicate that the miRNA-target pairs that mediate global sRNA and transcriptomic reprogramming play an important role in the morphological acclimation of the roots of *P.* × *canescens* in response to different N forms.

## Materials and methods

### Plant cultivation and N treatments

Plantlets of *P. tremula* × *P. alba* (INRA 717-IB4 clone) were obtained by the micropropagation method. This plant stock was obtained and granted by Prof. Andrea Polle’s laboratory at the University of Göttingen in Germany. Experimental research on these plants complied institutional and national guidelines. Seedlings were grown in a climate chamber for 30 days. Thereafter, the plants were placed in plastic pots (10 L) filled with fine sand and cultivated in a growing chamber with conditions similar to those of the climate chamber. Every other day, 50 mL of modified Long Ashton (LA) solution was provided to each plant for 14 days, with the modified LA solution configured according to P Dluzniewska et al [48]. Subsequently, the plants were transplanted into N-free medium for 3 days, a time determined by reference to the results of S Balazadeh et al [49]. The plants were then transferred to LA nutrient solution for 21 days of hydroponics, in which 500 μM NaNO_3_ in LA solution was replaced by 1 mM NaNO_3_ (S1), 500 μM NH_4_NO_3_ (S2) and 1 mM NH_4_Cl (S3). The nutrient solution was changed every other day. At each treatment level, eighteen plants showing similar growth performance were used for further study.

### Root measurements

Root lengths (length of taproots from the stem end to the root tip) were measured with a ruler. The root tip was compressed with a transparent agent (chloral hydrate: water: glycerin = 8 g: 2 ml: 1 ml) and observed and measured under a microscope. The location of lateral root initiation was measured under a microscope. The number of lateral roots within a range of 1 cm from the start site of lateral root initiation was counted under a microscope as an indicator of lateral root density. Six plants were measured at each treatment level, and three biological replicates were performed.

### Harvesting

The whole roots of each plant were carefully cleaned with a modified LA solution containing 1 mM NaNO_3_, 500 μM NH_4_NO_3_ and 1 mM NH_4_Cl. Each root sample was wrapped in aluminum foil and snap-frozen in liquid nitrogen. A ball mill (MM400, Retsch, Haan, Germany) was used to grind each root sample into fine powder, which was stored at -80 °C. The same amounts of fine powder were extracted from the root samples of six plants and mixed evenly to form a mixed sample. Thus, three mixed samples were obtained.

### sRNA RNA library construction and sequencing

Total RNA from the samples was isolated using TruSeq Small RNA Prep Kits (RS-200, Illumina, CA, USA) to obtain a small RNA library. Three small RNA libraries for each treatment level were constructed and sequenced on an Illumina HiSeq 2500 sequencer (Illumina, CA, USA) at LC Science (Hangzhou, China) according to the protocol recommended by the supplier. All of the small RNA sequences are available in the Sequence Read Archive (SRA) under project ID PRJNA631845.

### Small RNA data analysis and miRNA identification

Known and novel miRNAs were identified according to the procedure proposed by J Zhou et al [7]. Raw Illumina sequencing data were further processed using the ACGT101-miR program (LC Sciences, Houston, Texas, USA) to remove adapter dimers, junk sequences, sequences of low complexity, and common RNA family members (rRNA, tRNA and snRNA). Then, the remaining clean and unique reads with lengths of 18–25 nt were aligned to the sequences from the miRBase 22.0 database to identify known miRNAs, with one mismatch allowed per sequence. Novel miRNA candidates were identified based on their genome location and stem–loop hairpin structure, and secondary structures were predicted using RNAfold software as suggested by S Griffiths-Jones et al [50]. In this study, pre-miRNAs with a stable hairpin structure and a minimal folding free energy index (MFEI) greater than 0.9 were considered novel miRNAs.

The frequency of the miRNAs in each sample was normalized to transcripts per million (TPM) values. The log_2_(fold change) of the differentially expressed miRNAs was determined by the TPM ratio of S1 vs. S2 or S3 vs. S2. *P* values of less than 0.05 were considered significantly differentially expressed miRNAs. Cytoscape software (3.5.1) was used to construct heatmaps with significantly differentially expressed miRNAs. S1_1, S1_2 and S1_3 represent three biological repeats of S1, S2_1, S2_2 and S2_3 represent three biological repeats of S2, and S3_1, S3_2 and S3_3 represent three biological repeats of S3.

### Degradome sequencing and target identification

A total RNA purification kit (TRK1001, LianChuan (LC) Science, Hangzhou, China) was used to extract total RNA from the samples, and a NanoDrop 2000 spectrophotometer (Thermo, Wilmington, DE) was used to assess the RNA quality according to a 260/280 nm ratio > 2.0. Three independent RNA samples of equal quantities at the same treatment level were mixed to produce one degradation library. Then, three degradome samples (S1, S2 and S3) were sequenced on the Illumina HiSeq2500 platform (LC-BIO, Hangzhou, China). Degradome library construction was performed according to Xu X et al [51]. Data analysis was performed using CleaveLand 3.0 software with specific methods referred to C Addo-Quaye et al [52]. The degradome sequencing data were also submitted to the SRA under project ID PRJNA631839.

### Identification and annotation of differentially expressed target genes

To determine the expression profiles of the target genes, three cDNA libraries from each treatment were constructed from the RNA samples, and transcriptomic sequencing was performed on an Illumina HiSeq2000/2500 sequencer (Illumina) at Lianchuan Science (Hangzhou, China) according to the protocol recommended by the supplier. The transcriptomic sequencing data were also submitted to the SRA under project ID PRJNA631840.

For each library, all sequences were processed by filtering out adaptor sequences and low-quality sequences. Subsequently, all clean tags were mapped to unigenes assembled for *P.* × *canescens* for annotation, and the degradome reads were mapped according to the *P.* × *canescens* transcriptomic data (http://aspendb.uga.edu/index.php/databases/spta-717-genome). After the miRNA, degradome sequencing and transcriptomic sequencing data were combined as described by M Pertea et al [53], the fragments per kilobase of exon per million fragments mapped (FPKM) algorithm was used to calculate the target gene expression level. The log_2_(fold change) values of differentially expressed target genes were calculated using the FPKMs of genes determined under S1 vs. S2 or S3 vs. S2. Differentially expressed target genes with *P* values of less than 0.05 and absolute log_2_ (fold change) values greater than 1 were considered significantly differentially expressed target genes.

### Functional analysis of target genes

To clarify the functions of the target genes, a Gene Ontology (GO) functional classification [54] and KEGG (Kyoto Encyclopedia of Genes and Genomes) enrichment analysis [55] were conducted. MapMan was also used to analyze the pathways [56].

### Quantitative RT–PCR validation of significantly differentially expressed miRNAs and genes

To verify the significantly differentially expressed miRNAs and their target genes, the samples used for sequencing as described above were employed for qRT–PCR validation using the PrimeScript™ RT reagent Kit and TB Green qRT–PCR kit (Takara, Dalian, China) as suggested by J Zhou et al [12]. Thirty-seven significantly differentially expressed miRNAs and 12 significantly differentially expressed miRNA-target pairs were selected for qRT–PCR analysis. Each miRNA or target gene was analyzed in three replicates. The specific primers of mature miRNAs and their target genes are shown in Supplementary Table 1. Two genes, *U6* and *ACTIN2/7*, were selected as reference genes for the validation of miRNAs and target genes, respectively [12].

### Validation of miRNA-target pairs

To validate the cleavage of target genes by miRNAs, transient coexpression assays were performed using *Nicotiana benthamiana* leaves according to the methods of J Zhou et al [7]. Two selected miRNA-target pairs were chosen. In brief, fragments of the target genes were cloned and ligated into the pCAMBIA1300 vector for mRNA overexpression. Similarly, genomic DNA fragments encoding miRNA precursors were amplified using genomic DNA from root samples and sequence-specific primers (Supplementary Table 1). The amplified miRNA precursor fragments were inserted into the pCAMBIA2300 vectors for miRNA overexpression. Then, the two vectors were transformed into *Agrobacterium tumefaciens* strain GV3101 by electroporation. The Agrobacterial cell cultures were inoculated overnight at 28 °C. As previously described [57], equal amounts of Agrobacterial cell cultures containing miRNAs and their target genes were mixed and infiltrated into *N. benthamiana* leaves. After incubation in the dark for two days, the infiltrated leaves were harvested for qRT–PCR. analysis with gene-specific primers (Supplementary Table 1). Tubulin genes (Supplementary Table 1) served as the reference genes for target genes.

### Statistical analysis

Statistical analysis of the data was performed using Statgraphics software (STN, St Louis, MO, USA). Before statistical analysis, the data were tested for a normal distribution. All the data were analyzed by *one-way analysis of variance (ANOVA)*, and the different N forms tested were included as a factor. When the *P* value of the *ANOVA F* test was less than 0.05, the differences between the means were considered significant.

## Supplementary Information

**Additional file 1. **Supporting informationAdditionalsupporting information can be found in the online* version of this article: ***Supplementary Fig.1.**Lengths of total small RNAs (a) and unique small RNAs (b) in the S1, S2 and S3libraries.**Supplementary Fig.2. **Validation of significantly differentially expressed miRNAs. **Supplementary Fig. 3.**Analysis of GO functional classification of identified differentially expressedtarget genes. S1 vs. S2 (a) and S3 vs. S2 (b). **Supplementary Fig. 4.** KEGG pathway analysis of identified differentially expressed target genes. S1 vs. S2(a) and S3 vs. S2 (b).

**Additional  file 2:**  **Supplementary Table1.** Primers used for qRT–PCR.**Supplementary Table 2.** Distribution of smallRNAs in different categories.**Supplementary Table 3.**Identified known miRNAs.**Supplementary Table 4.**Identified miRNAfamilies.**SupplementaryTable 5.** Identified novel miRNAs.**Supplementary Table 6.**Significantly differentially expressed known and novelmiRNAs.**Supplementary Table 7.**Degradome sequencing forthe identification of target genes of known and novel miRNAs.**Supplementary Table 8.** Target genes ofsignificantly differentially expressed miRNAs.**Supplementary Table 9.**Annotated analysis of the significantly differentially expressed target genesof DEmiRNAs.

## Data Availability

All of the small RNA sequences are available in the Sequence Read Archive (SRA) under project ID PRJNA631845 (https://www.ncbi.nlm.nih.gov/bioproject/PRJNA631845). The degradome sequencing data were also submitted to the SRA under project ID PRJNA631839 (https://www.ncbi.nlm.nih.gov/bioproject/PRJNA631839). The transcriptomic sequencing data were also submitted to the SRA under project ID PRJNA631840 (https://www.ncbi.nlm.nih.gov/bioproject/PRJNA631840).
