# Supplementary material for: Nitrate/ammonium-responsive microRNA-mRNA regulatory networks affect root system architecture in Populus × canescens
Source: BMC Plant Biol. 2022 Mar 4;22:96. doi: 10.1186/s12870-022-03482-3 (PMC8895855; doi:10.1186/s12870-022-03482-3)
Supplement: Supplementary file 1 — Additional file 1. Supporting informationAdditionalsupporting information can be found in the online version of this article: Supplementary Fig.1.Lengths of total small RNAs (a) and unique small RNAs (b) in the S1, S2 and S3libraries.Supplementary Fig.2. Validation of significantly differentially expressed miRNAs. Supplementary Fig. 3.Analysis of GO functional classification of identified differentially expressedtarget genes. S1 vs. S2 (a) and S3 vs. S2 (b). Supplementary Fig. 4. KEGG pathway analysis of identified differentially expressed target genes. S1 vs. S2(a) and S3 vs. S2 (b). [file 12870_2022_3482_MOESM1_ESM.docx]

**Supporting information**
Additional supporting information can be found in the online version of this article:

**Supplementary Fig. 1** Lengths of total small RNAs (a) and unique small RNAs (b) in the S1, S2 and S3 libraries.

**Supplementary Fig. 2** Validation of significantly differentially expressed miRNAs.

**Supplementary Fig. 3** Analysis of GO functional classification of identified differentially expressed target genes. S1 vs. S2 (a) and S3 vs. S2 (b).

**Supplementary Fig. 4** Analysis of KEGG pathway of identified differentially expressed target genes. S1 vs. S2 (a) and S3 vs. S2 (b).
