# Supplementary material for: Nitrate/ammonium-responsive microRNA-mRNA regulatory networks affect root system architecture in Populus × canescens
Source: BMC Plant Biol. 2022 Mar 4;22:96. doi: 10.1186/s12870-022-03482-3 (PMC8895855; doi:10.1186/s12870-022-03482-3)
Supplement: Supplementary file 2 — Additional file 2: Supplementary Table1. Primers used for qRT–PCR.Supplementary Table 2. Distribution of smallRNAs in different categories.Supplementary Table 3.Identified known miRNAs.Supplementary Table 4.Identified miRNAfamilies.SupplementaryTable 5. Identified novel miRNAs.Supplementary Table 6.Significantly differentially expressed known and novelmiRNAs.Supplementary Table 7.Degradome sequencing forthe identification of target genes of known and novel miRNAs.Supplementary Table 8. Target genes ofsignificantly differentially expressed miRNAs.Supplementary Table 9.Annotated analysis of the significantly differentially expressed target genesof DEmiRNAs. [file 12870_2022_3482_MOESM2_ESM.docx]

**Supplementary Table 1** Primers used for qRT–PCR.

**Supplementary Table 2** Distribution of small RNAs in different categories.

**Supplementary Table 3** Identified known miRNAs.

**Supplementary Table 4** Identified miRNA families.

**Supplementary Table 5** Identified novel miRNAs.

**Supplementary Table 6** Significantly differentially expressed known and novel miRNAs.

**Supplementary Table 7** Degradome sequencing for the identification of target genes of known and novel miRNAs.

**Supplementary Table 8** Target genes of significantly differentially expressed miRNAs.

**Supplementary Table 9** Annotated analysis of the significantly differentially expressed target genes of DEmiRNAs.
